# Germany’s Burden of Disease of Bloodstream Infections Due to Vancomycin-Resistant *Enterococcus faecium* between 2015–2020

**DOI:** 10.3390/microorganisms10112273

**Published:** 2022-11-16

**Authors:** Simon Brinkwirth, Sofie Martins, Olaniyi Ayobami, Marcel Feig, Ines Noll, Benedikt Zacher, Tim Eckmanns, Guido Werner, Niklas Willrich, Sebastian Haller

**Affiliations:** 1 Unit 37: Healthcare-Associated Infections, Surveillance of Antibiotic Resistance and Consumption, Department of Infectious Disease Epidemiology, Robert Koch Institute, Seestr. 10, 13353 Berlin, Germany; 2 Postgraduate Training for Applied Epidemiology (PAE), Robert Koch Institute, 13353 Berlin, Germany; 3 European Programme for Intervention Epidemiology Training (EPIET), European Centre for Disease Prevention and Control (ECDC), 16973 Solna, Sweden; 4 CP3-Origins & IMADA, University of Southern Denmark, Campusvej 55, DK-5230 Odense, Denmark; 5 Unit IT4: Development, Department of Methods Development, Research Infrastructure and Information Technology, Robert Koch Institute, Seestr. 10, 13353 Berlin, Germany; 6 Unit 32: Surveillance and Electronic Reporting and Information System (DEMIS), Department of Infectious Disease Epidemiology, Robert Koch Institute, Seestr. 10, 13353 Berlin, Germany; 7 Unit 13: Nosocomial Pathogens and Antibiotic Resistances, National Reference Centre for Staphylococci and Enterococci, Department of Infectious Diseases, Robert Koch Institute, Burgstr. 37, 38855 Wernigerode, Germany

**Keywords:** burden of disease, bloodstream infection, antimicrobial resistance, vancomycin-resistance, Enterococcus faecium, VRE, VREfm, DALYs

## Abstract

In Germany, there is an increasing amount of vancomycin-resistant *Enterococcus faecium* (VREfm) isolates in bloodstream infections (BSIs); however, estimates on recent incidences and disease burden are missing. We aim to estimate the incidence and calculate the annual disease burden in disease-adjusted life years (DALYs) for BSIs due to VREfm in Germany between 2015 and 2020 to support informed decision-making in the field of antimicrobial resistance (AMR). We used the Antibiotic Resistance Surveillance (ARS) system data to obtain incidence estimates. The estimated incidences were used in the *Burden of Communicable Disease in Europe* (BCoDE) toolkit to calculate the attributable DALYs. A total of 3417 VREfm blood culture-positive isolates were observed within ARS. The estimated incidence of VREfm-BSIs per 100,000 inhabitants increased from 1.4 (95% Uncertainty Interval [UI]: 0.8–1.9) in 2015 to 2.9 (95% UI: 2.4–3.3) in 2020. The estimated burden, expressed in DALYs per 100,000 inhabitants, increased from 8.5 (95% UI: 7.3–9.7; YLD = 0.9, YLL = 7.6) in 2015 to 15.6 (95% UI: 14.6–16.6; YLD = 1.6, YLL = 14) in 2020. The most affected groups within the observed period are the 65–69-year-old males with 262.9 DALYs per 100,000 inhabitants, and in the younger age groups (<30 years), the under-one-year-old with 43.1 DALYs per 100,000 inhabitants and 34.5 DALYs for male and female, respectively. The increasing DALYs of BSIs due to VREfm require targeted prevention and control measures to address their unequal distribution across gender and age, especially for older hospitalized patients, neonates, and infants in Germany.

## 1. Introduction

Antibiotic-resistant bacterial infections pose a threat to human health and modern health care in general. They are now widely acknowledged as a major threat to global health and the economy, with an estimated 1.27 million deaths attributable to AMR in 2019 [1,2]. Even though lower- and lower-middle-income countries are affected most severely, there is also a considerable burden across the member states of the European Union (EU) and European Economic Area (EEA) [3]. An accurate estimation of the current burden with the latest trends of infections differentiated by pathogens is missing, but it is needed to support informed decision-making by public health experts and policymakers. Since resources are limited, such estimates can effectively prioritize and help to plan targeted infection-prevention measures.

To fully capture the impact of disease, disease-adjusted life years (DALYs) were introduced as a composite metric, taking years of life lost (YLL) and years lived with disease (YLD) into account to measure the burden of disease [4,5]. DALYs allow for a comparison of different diseases with different severity in order to prioritise public health measures. The burden of HAIs in Europe was estimated in 2016 by the European Centre for Disease Control and Prevention (ECDC), using the Burden of Communicable Disease in Europe (BCoDE) project approach [6,7]. Based on an adaptation of this approach, it was found that bloodstream infections (BSIs) accounted for the highest number of attributable deaths compared to other identified healthcare-associated infections (HAIs) and a high burden of disease in comparison to other infectious diseases [8].

One of the most frequently isolated pathogens in BSIs is *Enterococcus faecium* [9,10]. *Enterococcus faecium* is of high clinical relevance due to its low natural susceptibility to multiple antibiotics, including low-dose penicillin and ampicillin, aminoglycosides, cephalosporins, and sulphonamides [11,12]. In the last decade, the proportions of vancomycin resistance among *Enterococcus faecium* isolates in Germany and the EU/EEA have been rising [13,14,15,16]. Therefore, we aim to estimate the disease burden in DALYs attributable to BSIs due to vancomycin-resistant *Enterococcus faecium* in Germany, using *Antibiotic Resistance Surveillance* (ARS) data from 2015 to 2020 and the BCoDE toolkit [17,18].

## 2. Materials and Methods

The burden calculation of VREfm in Germany proceeded in two steps. First, we estimated the incidence per 100,000 inhabitants of VREfm-BSIs, stratified by age and gender, from 2015 to 2020 based on data from the ARS surveillance system [17]. Second, based on the estimated incidences, the BCoDE toolkit [18] was used to estimate the annual burden of BSIs due to VREfm in DALYs per 100,000 inhabitants.

### 2.1. Study Data

The ARS data was used to estimate VREfm incidences in Germany. ARS is a laboratory-based voluntary surveillance system developed by the Robert Koch Institute. It contributes German surveillance data on antimicrobial resistance to the European Antimicrobial Resistance Surveillance Network (EARS-Net) and the Global Antimicrobial Resistance Surveillance System (GLASS). In 2020, 57 laboratories submitted data from 552 general hospitals. The participating laboratories supply data on routine diagnostics of a broad range of clinically relevant pathogens [17], including *Enterococcus faecium*. Trends in participating hospitals with isolates (not necessarily *Enterococcus faecium*) in the surveillance system for 2015 to 2020 and the distribution of the hospital type stratified by region differed between the observed period and are presented in the supplement (Figures S1 and S2). The most recent data of the surveillance system are shared in an aggregated form via interactive reports on the project website [19].

### 2.2. Outcome Definition, Variables of Interest, and Selection of Isolates

Only VREfm-positive blood cultures sent in by general hospitals were considered for our calculations. Further, only the first positive blood culture per quarter for each patient, as identified in the surveillance system by a per-lab unique identifier, was taken into account. For the purpose of the study, all VREfm blood culture-positive isolates in ARS with the pre-stated restrictions were considered VREfm-BSI. These positive blood cultures include isolates from both primary and secondary BSIs. Vancomycin resistance was defined according to the laboratories’ classifications based on the CLSI and EUCAST antimicrobial susceptibility testing guidelines. The data set includes demographic (*age* and *sex* of patient), clinical (*date of explant of the specimen* and *hospital type),* and geographical (*federal state of organisation submitting the isolate*) data. The age variable was grouped into 5-year steps except for <1-year-olds, 1 to 4-year-olds, and those older than 85 years. The age grouping is necessary for the predefined input layout of the BCoDE toolkit. Geographical regions were defined according to the ARS dataset: *North West* (Bremen, Hamburg, Lower Saxony, and Schleswig-Holstein)*, West* (North Rhine-Westphalia)*, South West* (Baden-Württemberg, Hesse, Rhineland-Palatinate, and Saarland)*, South East* (Bavaria, Saxony, and Thuringia) and *North East* (Berlin, Brandenburg, Mecklenburg-Western Pomerania, and Saxony-Anhalt).

### 2.3. Estimation of Incidences of BSIs Due to VREfm

To estimate incidences of BSIs due to VREfm, we used a survey design stratifying by region and year. Hospitals were treated as primary sampling units to account for cluster effects. Further, we estimated the coverage of the ARS surveillance system by the ratio of all general hospitals reporting complete data for the whole year in ARS for the respective region and year and the total number of general hospitals given by the official hospital statistics of Germany [20]. Our survey design used these estimated coverage values as sampling probabilities. Considering the coverage and the cluster effects of sampling whole hospitals, we estimated the incidences of VREfm-BSIs stratified by age and gender as necessary for applying the BCoDE methodology. To account for missing values in the gender variable, we added a correction factor to the incidence estimates, which accounted for the proportion of missing values. We used R (version 4.1.2) and the *survey* package to obtain the incidence estimates [21,22].

### 2.4. Estimation of DALYs

DALYs consist of two separate contributions: DALY = YLL + YLD. The first term YLL represents the years of life lost due to premature mortality caused by the disease or one of its sequelae. The years of life lost are calculated based on standard life expectancies. The second term YLD represents the years lived with the disease weighted by a disease weight specific to each disease or sequela. The disease weights aim to reflect the extent of the loss in quality of life attributable to the underlying disease or the sequela [4].

To estimate DALYs for BSIs due to VREfm, we followed the approach of the BCoDE project. It uses an incidence- and pathogen-based method for measuring infection burden, considering the infection’s direct disease outcome and sequelae [7,23]. The approach was already applied to the burden of antibiotic-resistant bacteria in the EU and EEA [3]. The paper included a disease model describing the probabilities for the different disease outcomes and sequelae for BSIs due to vancomycin-resistant *Enterococcus faecalis* and *Enterococcus faecium* shown in the supplement (Figure S3). We used the disease model to calculate the unique burden for BSIs due to VREfm since it is one of the most frequently isolated pathogens in BSIs. Further, vancomycin resistance is predominantly found in *Enterococcus faecium* (12–25.8%, 3417 isolates) and barely in *Enterococcus faecalis* (0,1%, 22 isolates) in Germany within the observed period 2015–2020 in ARS [19]. Therefore, *Enterococcus faecalis* isolates are excluded from the calculation.

We used the BCoDE toolkit version 2.0.0 provided by the ECDC [18] with *n* = 1000 simulations and no time discount. Based on the disease model and the incidences, the BCoDE toolkit runs Monte Carlo simulations to incorporate the uncertainties of the model parameters and the incidences in our results, which allow us to give uncertainty intervals (UIs) for our estimates. Uncertainty on the incidence estimates stratified by age group and sex were considered using the upper and lower boundaries of their 95% confidence intervals as the maximum and minimum of a PERT distribution which was taken as input for the BCoDE model.

To allow comparisons with previous publications, we used the standardised life expectancy table from the Global Burden of Disease project [3,6]. Age- and sex-stratified population numbers for Germany for 2015–2020 were used for our analyses [3].

## 3. Results

Between 2015 and 2020, a total of 3417 VREfm blood culture-positive isolates (female = 1082; male = 1926; unknown = 409) were observed within the ARS surveillance system. The total number of VREfm-BSIs increased from 209 (female = 73; male = 126; unknown = 10) in 2015 to 868 (female = 282; male = 486; unknown = 100) in 2020.

Extrapolating the ARS data to all German hospitals, the number of VREfm-BSIs is estimated to be 1115 in 2015 and 2345 in 2020. Based on the extrapolating data, the estimated incidence of VREfm-BSIs per 100,000 inhabitants increased from 1.4 (95% UI: 0.8–1.9) in 2015 to 2.9 (95% UI: 2.4–3.3) in 2020. The estimated incidence more than doubled within this period.

A total of 2499 attributable deaths (95% UI: 2311–2690) due to VREfm-BSIs were estimated by our model within this period. Over time, the estimated attributable deaths more than doubled from 2015 with 265 deaths (95% UI: 231–301) to 2020 with 557 deaths (95% UI: 524–586).

**Figure 1:**
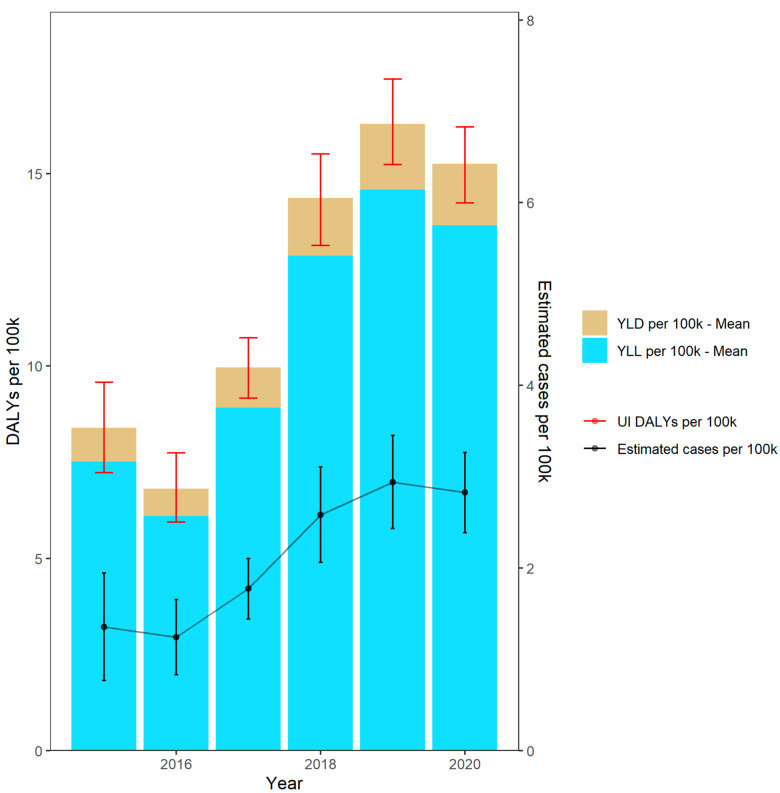
DALYs per 100,000 inhabitants and estimated incidence of bloodstream infections due to vancomycin-resistant *Enterococcus faecium* between 2015 and 2020 in Germany. UI: Uncertainty Intervals; YLL: Years of life lost; YLD: Years lived with disease.

The estimated annual burden, expressed in DALYs per 100,000 inhabitants, increased from 8.5 (95% UI: 7.3–9.7) in 2015 to 15.6 (95% UI: 14.6–16.6) in 2020 (Figure 1). In 2016 and 2020, a decline in the DALYs was detected compared to the previous year. However, especially between 2017 and 2019, a marked increase of 6.53 DALYs was observed, with consistently growing numbers of observed isolates throughout the years. The observed increase in DALYs included a rise in the YLLs from 7.6 (95% UI: 6.5–8.7) in 2015 to 14 (95% UI: 13.1–14.8) in 2020, as well as for the YLDs from 0.9 (95% UI: 0.8–1.0) in 2015 to 1.6 (95% UI: 1.5–1.8) in 2020, per 100,000 inhabitants. The estimated incidence and DALY trends of BSIs due to VREfm increased between 2015 and 2019, while the latest development from 2018 to 2020 points towards a flattened trend.

### 3.1. DALYs Stratified by Region

**Figure 2:**
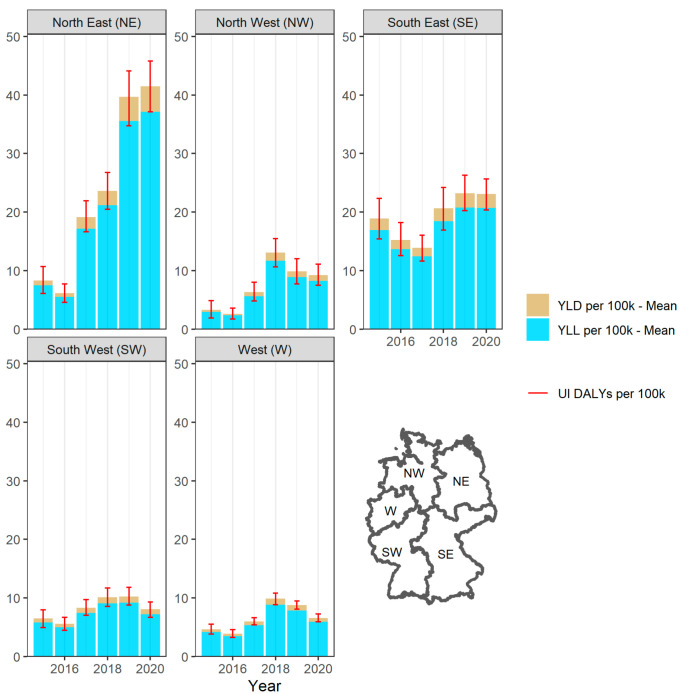
DALYs of bloodstream infections due to vancomycin-resistant *Enterococcus faecium* per 100,000 inhabitants, stratified by regions between 2015 and 2020 in Germany. UI: Uncertainty Intervals; YLL: Years of life lost; YLD: Years lived with disease.

The accumulated burden of VREfm-BSIs is not equally distributed across Germany (Figure 2). We found an increase of the total burden in DALYs per 100,000 inhabitants between 2015 and 2020 from 4.6 (95% UI: 3.8–5.5; YLD = 0.5, YLL = 4.1) to 6.6 (95% UI: 5.9–7.3; YLD = 0.7, YLL = 5.9) and 3.3 (95% UI: 1.9–4.9; YLD = 0.4, YLL = 2.9) to 9.2 (95% UI: 7.5–11.1; YLD = 1, YLL = 8.2) for the *West* and *North West* regions, respectively. Regarding the latest developments, both regions indicate a decreasing trend; overall, they are the least affected regions. The annual burden in the *South West* increased from 6.5 (95% UI: 4.9–7.9; YLD = 0.7, YLL = 5.8) to 8.1 (95% UI: 6.7–9.3; YLD = 0.8, YLL = 7.2) between 2015 and 2020. The *South West* is stronger impacted compared to the *West* and *North West* but based on the latest decline, no clear trend in the development is visible. The *South East* region recorded higher estimated DALYs within the observed years ranging from 18.9 (95% UI: 15.4–22.3; YLD = 2, YLL = 16.9) to 23.1 (95% UI: 20.4–25.7; YLD = 2.4, YLL = 20.7) per 100,000 inhabitants. In the *North East*, the DALYs increased quite consistently from 8.3 (95% UI: 6.1–10.7; YLD = 0.9, YLL = 7.4) to 41.5 (95% UI: 37.1–45.8; YLD = 4.3, YLL = 37.2) per 100,000 inhabitants and represents the most impacted region regarding the disease burden. The *North East* and *South East* still indicate an increasing trend over the whole period but a flattened trend addressing the last two years.

### 3.2. DALYs Stratified by Age and Sex

**Figure 3:**
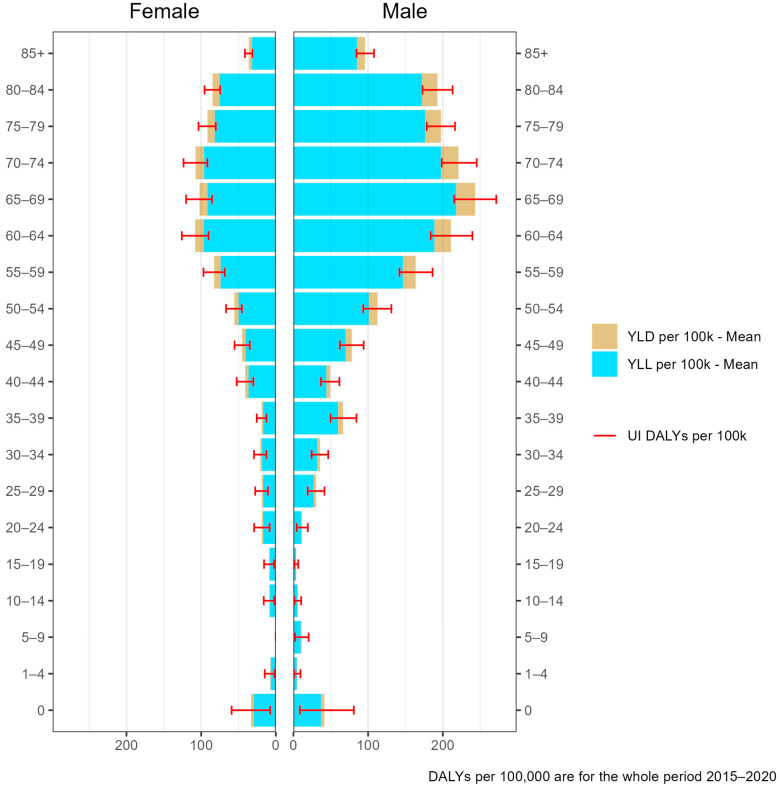
DALYs per 100,000 inhabitants stratified by age and sex for the whole study period in Germany (2015–2020). UI: Uncertainty Intervals; YLL: Years of life lost; YLD: Years lived with disease.

Men were identified with a higher burden due to VREfm-BSIs with 93.4 DALYs (95% UI: 88.9–98.0; YLD = 9.8, YLL = 83.6) compared to women with 50.0 DALYs (95% UI: 47.1–52.8; YLD = 5.3, YLL = 44.7) per 100,000 inhabitants for the observed aggregated period from 2015–2020 in Germany (Figure 3). This higher burden share among men was observed annually from 2015 to 2020. Stratified by age, the same pattern is observed among patients above the age of 30 years. For the total burden due to VRE-BSIs, the age group 65–69 with 262.9 DALYs (95% UI: 232.5–293.3; YLD = 27.6, YLL = 235.2) and 110.5 DALYs (95% UI: 92.3–129.9; YLD = 11.5, YLL = 99) for male and female, per 100,000 inhabitants, respectively, were affected most (Figure 3). The burden was generally low for those under 30 years, and no clear discernible patterns by sex or age were observed. In the younger age groups (<30 years), under-one-year-olds are affected the most with 43.1 DALYs (95% UI: 8.9–84.2; YLD = 4.4, YLL = 38.7) and 34.5 DALYs (95% UI: 7.8–61.5; YLD = 3.6, YLL = 30.9) for males and females, respectively.

## 4. Discussion

In this study, we report increasing incidences and DALYs of BSIs due to VREfm in German hospitals between 2015 and 2020 using a population-based quantification based on 3417 VREfm blood culture-positive isolates reported in ARS. The estimated incidences of VREfm-BSIs per 100,000 inhabitants increased from 1.4 to 2.9, with a total of 2499 estimated attributable deaths within the whole study period. The annual burden, expressed in DALYs per 100,000 inhabitants, increased from 8.5 (YLL = 7.6; YLD = 0.9) in 2015 to 15.6 (YLL = 14; YLD = 1.6) in 2020. However, an east–west gradient for the annual burden and the latest trends were found with higher DALYs in Germany’s *North East* and *South East* regions. Across all regions, men in the older age groups were more affected, as well as the under-one-year-old for the younger age groups. Differences in the estimated DALYs per region, sex, and age group are predominantly explained by the different incidence levels. This finding buttresses the need for gender- and age-stratified testing for relevant pathogens, as previously shown for other priority infections in Germany [24].

The occurrence and distribution of *E. faecium* and *E. faecalis* in enterococcal infections and their vancomycin resistance varies widely and can therefore cause heterogenous incidences and disease burden [13,25]. Our specific calculation for VREfm provides an appropriate estimation of the burden, since vancomycin resistance was predominantly found in *Enterococcus faecium* in Germany from 2015 to 2020 [19].

With 8.5 DALYs per 100,000 inhabitants in 2015, the burden of VREfm in Germany seems higher than the EU/EEA average estimates for vancomycin-resistant *Enterococcus* spp. (5.5 DALYs per 100,000 inhabitants) in 2015 [3]. However, it remains challenging to make direct comparisons due to differences in the methodological approaches. Even though there are, to our knowledge, no published estimates on DALYs attributable to VREfm infections in Europe after 2015, the increasing trend in the proportion of VREfm in Germany [15] in the last few years mirrored the trend in Europe even before the COVID-19 Pandemic [13,26]. Over time, Germany’s DALYs attributable to VREfm almost doubled from 2015 to 2020. In the same period, there was a rise in the total amount of isolates of *Enterococcus faecium* and a corresponding rise in the resistance to vancomycin, observed in Germany and the EU/EEA [13,15].

The general high variation of the incidence of infections due to VREfm is a phenomenon detected between countries, regions, and even among hospitals within a region across Europe [9]. The observed regional difference of the DALYs attributable to VREfm infections in this study is in line with the prevalence and occurrence of VREfm within the last few years in Germany [15]. It is important to note that the observed differential burden seems to depend on the region’s demographic structure and the distribution of care facility levels sampled in ARS (Figure S2). Therefore, interpretation should prioritize the different regional trends rather than directly comparing the total number of DALYs between regions.

In our dataset, there was an observable difference in the disease burden between male and female patients. This observation is in line with previous studies reporting gender differences in the occurrence of specific AMR-related pathogens [6,24]. This finding might be explained by the higher male share of the underlying population at risk for BSIs in Germany since males account for more hospital cases and hospital days, especially those aged 50 and above [27]. Still, Brandl et al. showed—for some AMR-related pathogens—that gender differences remained when correcting for age and hospital stays [24]. Limited evidence has implicated genetic and hormonal differences as contributory factors that mediate differences in susceptibility to infections, including resistant infections, between men and women [28,29].

As previously observed in different studies across Europe, the older groups (age groups >50 years) bear the majority of the disease burden of bacterial infections, including the resistant variants [6,30]. This is not surprising, considering older people are more likely to have waning immunity, a history of previous hospitalization, and exposure to multiple antibiotics uses during their life course than younger populations [31]. Besides the older age group, the under-one-year-old group is impacted the most among the younger age groups. The vulnerability and occurrence of VREfm in this age group are frequently observed in outbreak investigations and associated with additional risk factors, such as a NICU stay, immunosuppression, low birth weight, and other underlying diseases [32,33].

Several limitations of the study need to be addressed for balanced interpretation. The dataset does not include any strain typing data. This might be relevant in a situation of a changing epidemiology of occurring epidemic strains during the study period or a regional clustering of VRE clones, where the divergently prevalent strain types might exhibit different levels of pathogenic potential [34].

Even though the ARS surveillance system provides reliable data for the given calculations, it is voluntary surveillance, which affects the data set’s representativeness, and the coverage and mix (in terms of care level) of included hospitals differ between the regions and between years. Especially in the first two years of the study period, there is still an observable change in the mix of hospital care levels and the numbers of participating hospitals across regions (Figures S1 and S2). Furthermore, while aggregating several federal states into regions was necessary to obtain a large enough sample of hospitals in each region for robust incidence estimates, it can be more challenging to observe trends on the level of individual federal states. As health policy is often decided at the federal-state level in Germany, detecting such trends would be highly desirable.

Within the dataset, only limited epidemiological information on the patients is available. Therefore, in-depth analyses addressing, e.g., different employment sectors or other potential risk factors are not possible.

Furthermore, the case mix and specializations of hospitals included in the analyses are not considered, and infections due to multiple organisms are included in the calculation. In this estimation, a sample of the less healthy population (hospital population) was used to draw conclusions for the general population, while adjustments for comorbidities and life expectancy were not captured. The advantages and disadvantages of different choices for life expectancy tables in burden studies are discussed in the literature [35]. Last, the BCoDE model for vancomycin-resistant *Enterococcus faecium* and *Enterococcus faecalis* was used to calculate only the burden of vancomycin-resistant *Enterococcus faecium*.

Despite these limitations, our study provides a robust estimation of the burden of VREfm. The approach pioneered for VRE in this study could be extended to study other country-specific burdens of pathogens in the future, including pathogens with and without AMR. Additionally, to our knowledge, this is the first study calculating the burden of disease for BSIs due to VREfm stratified by age, sex, and region for Germany.

## 5. Conclusions

The increasing DALYs of BSIs due to VREfm reflect the ongoing adverse impact of antimicrobial-resistant infections on the healthy life years of patients in Germany, in particular, elder hospitalized men, neonates, and infants. These findings provide a compelling basis for targeted bacterial infection and prevention measures and should guide policy priorities for strengthened AMR surveillance in Germany.

## Data Availability

The most recent data of the ARS surveillance system are shared in an aggregated form via interactive reports on the project website: https://ars.rki.de/ (accessed on 10 October 2022).

## Supplementary Materials

The following supporting information can be downloaded at: https://www.mdpi.com/2076-2607/10/11/2273/s1. Figure S1: Number of general hospitals and coverage of general hospitals of ARS by region. Figure S2: Distribution of Healthcare Level of hospitals in ARS per Year and Region. L1—smaller general hospitals (<200 beds), L2—larger general hospitals (200–800 beds) with less than 10 specialized units, L3—larger general hospitals (200–800 beds) with more than 10 specialized units, L4—largest general hospitals (>800 beds) and university hospitals, L5—specialized hospitals (e. g. eye clinics, etc.). Figure S3: Disease Outcome Tree: Enterococcus faecalis and Enterococcus faecium VRE BSI Model-Germany. CI: Cognitive impairment; PI: Physical impairment; PTSD: Post-traumatic stress disorder; R: Recovered; RF: Renal failure, renal replacement. The transition probabilities of the outcome tree are drawn from a PERT distribution to account for the uncer-tainty of parameter estimates. The values shown in parentheses are the most likely values of the used PERT distributions. For the complete parametrisation, see [18].

## Abbreviations

AMR: Antimicrobial Resistance; ARS: Antibiotic Resistance Surveillance; BCoDE: Burden of Communicable Disease in Europe; CDC: Centers for Disease Control and Prevention; DALYs: Disease-adjusted Life Years; ECDC: European Centre for Disease Control and Prevention; EEA: European Economic Area; EU: European Union; BSIs: Bloodstream Infections; HAIs: Healthcare-associated Infections; UI: Uncertainty Interval; VREfm: Vancomycin-Resistant *Enterococcus faecium*; YLD: Years Lived with Disease; YLL: Years of Life Lost.
