# Supplementary material for: Germany’s Burden of Disease of Bloodstream Infections Due to Vancomycin-Resistant Enterococcus faecium between 2015–2020
Source: Microorganisms. 2022 Nov 16;10(11):2273. doi: 10.3390/microorganisms10112273 (PMC9717732; doi:10.3390/microorganisms10112273)
Supplement: Supplementary file 1 [file microorganisms-10-02273-s001.zip › microorganisms-1994171-supplementary.pdf]

microorganisms

MDPI

Supplement: Germany's Burden of Disease of Bloodstream Infections Due to Vancomycin-Resistant *Enterococcus faecium* between 2015–2020

Simon Brinkwirth [1,2,*,†], Sofie Martins [3], Olaniyi Ayobami [1], Marcel Feig [4], Ines Noll [1], Benedikt Zacher [5], Tim Eckmanns [1], Guido Werner [6], Niklas Willrich [1,†] and Sebastian Haller [1,*,†]

1 Robert Koch Institute, Department of Infectious Disease Epidemiology, Unit 37: Healthcare-Associated Infections, Surveillance of Antibiotic Resistance and Consumption, Seestr. 10, 13353 Berlin, Germany
2 Postgraduate Training for Applied Epidemiology (PAE), Robert Koch-Institute, Berlin, Germany affiliated with the European Programme for Intervention Epidemiology Training (EPIET), European Centre for Disease Prevention and Control (ECDC), Solna, Sweden
3 CP3-Origins & IMADA, University of Southern Denmark, Campusvej 55, DK-5230 Odense M, Denmark
4 Robert Koch Institute, Department of Infectious Disease Epidemiology, Unit IT4: Development, Seestr. 10, 13353 Berlin, Germany
5 Robert Koch Institute, Department of Infectious Disease Epidemiology, Unit 32: Surveillance and Electronic Reporting and Information System (DEMIS), Seestr. 10, 13353 Berlin, Germany
6 Robert Koch Institute, Department of Infectious Diseases, Unit 13: Nosocomial Pathogens and Antibiotic Resistances, National Reference Centre for Staphylococci and Enterococci, Burgstr. 37, 38855 Wernigerode, Germany
* Correspondence: brinkwirths@rki.de (S.B.); hallers@rki.de (S.H.)
† These authors contributed equally to this work.

Table of Content

Figure S1 Number of general hospitals and coverage of general hospitals of ARS by region. 2

Figure S2 Distribution of Healthcare Level of hospitals in ARS per Year and Region. 3

Figure S3 Disease Outcome Tree: Enterococcus faecalis and Enterococcus faecium VRE BSI Model - Germany. 4

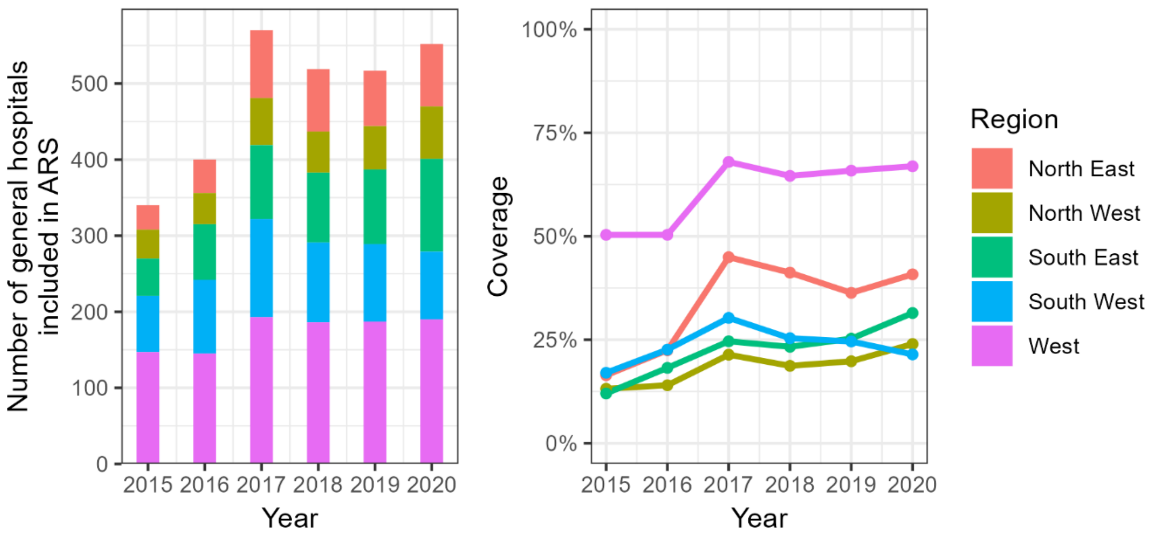

Figure S1. Number of general hospitals and coverage of general hospitals of ARS by region.

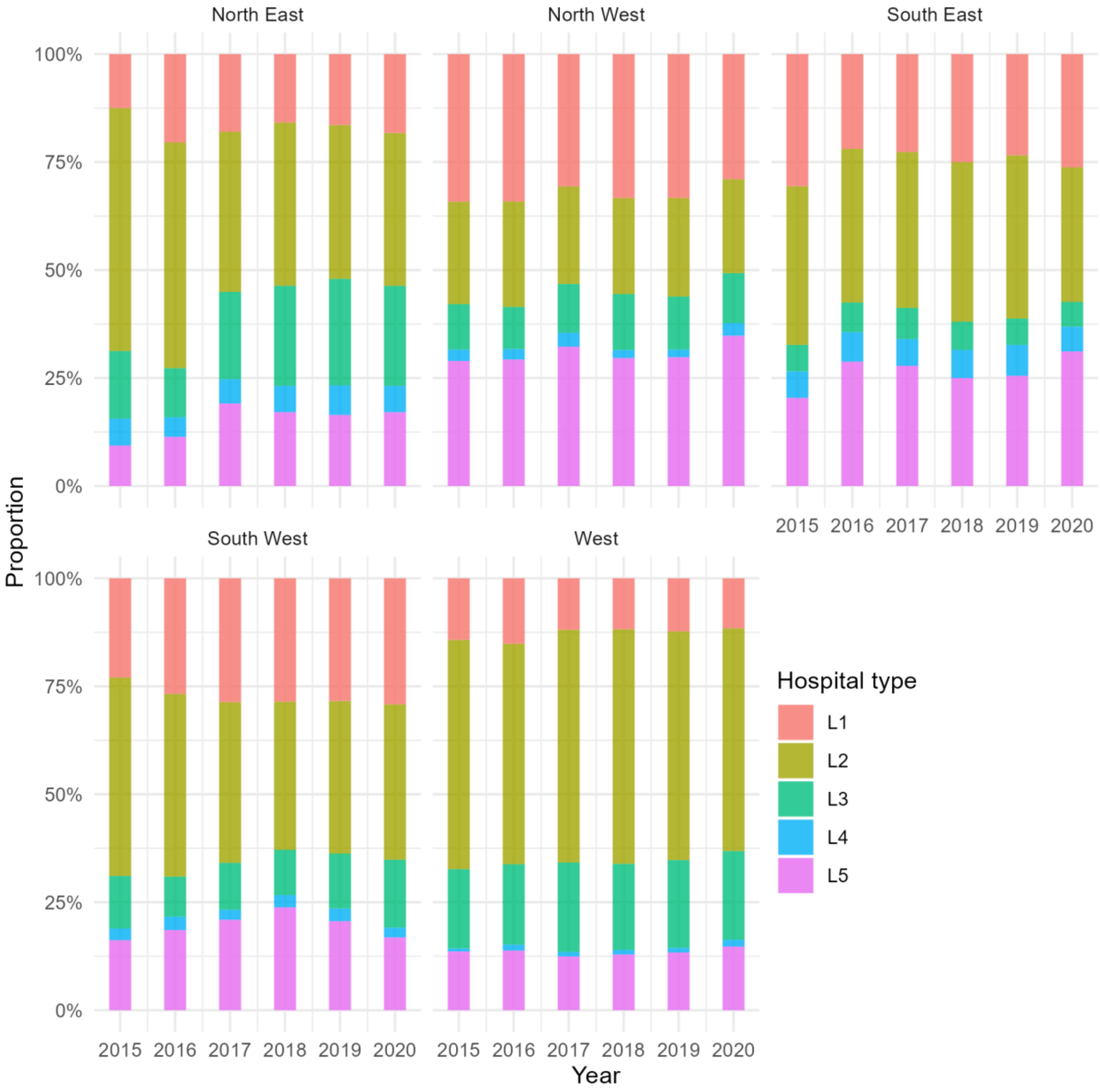

Figure S2. Distribution of Healthcare Level of hospitals in ARS per Year and Region. L1 - smaller general hospitals (<200 beds), L2 – larger general hospitals (200-800 beds) with less than 10 specialized units, L3 – larger general hospitals (200-800 beds) with more than 10 specialized units, L4 – largest general hospitals (>800 beds) and university hospitals, L5 – specialized hospitals (e. g. eye clinics, etc.).

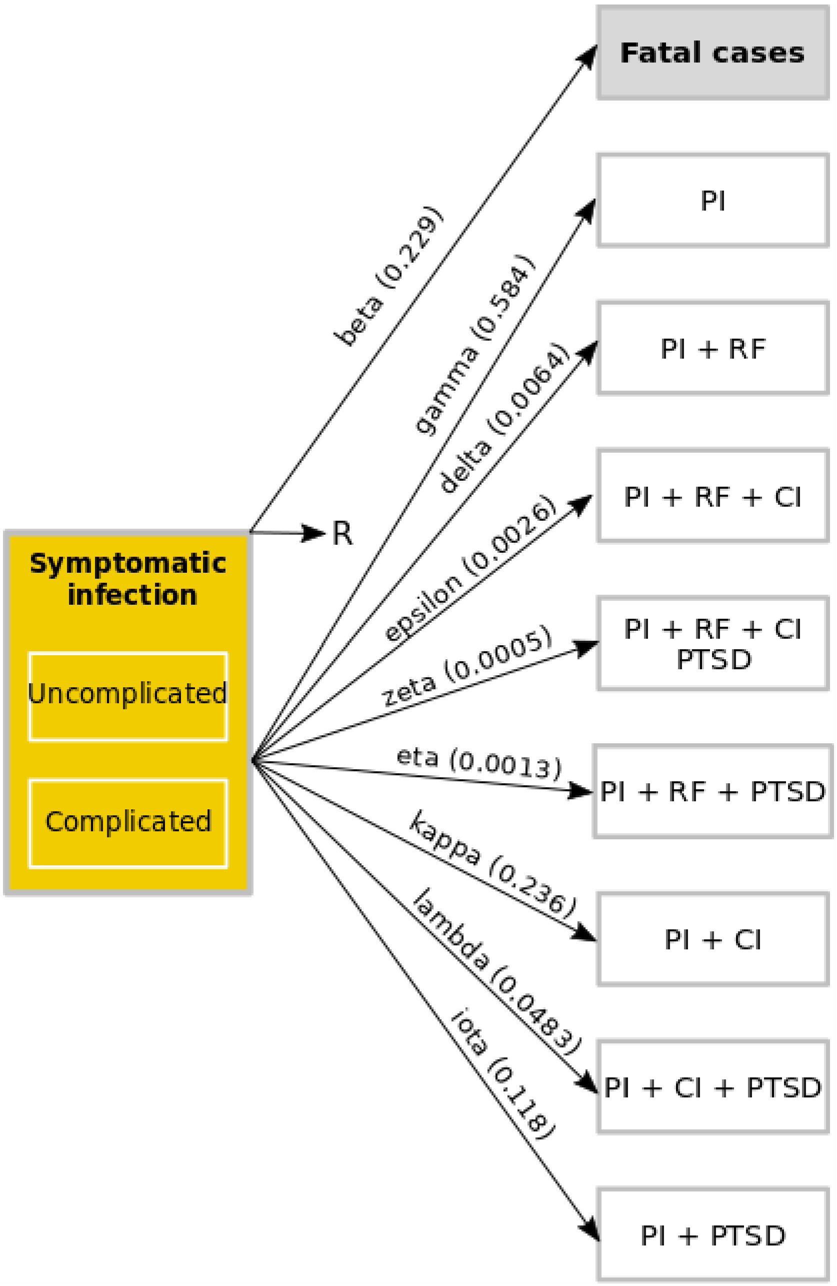

Figure S3. Disease Outcome Tree: Enterococcus faecalis and Enterococcus faecium VRE BSI Model - Germany. CI: Cognitive impairment; PI: Physical impairment; PTSD: Post-traumatic stress disorder; R: Recovered; RF: Renal failure, renal replacement. The transition probabilities of the outcome tree are drawn from a PERT distribution to account for the uncertainty of parameter estimates. The values shown in parentheses are the most likely values of the used PERT distributions. For the complete parametrisation, see (1).

1. European Centre for Disease Prevention and Control. ECDC BCoDE toolkit [software application]: Version 2.0.0 2020 [Available from: https://www.ecdc.europa.eu/en/publications-data/toolkit-application-calculate-dalys.
